# 非小细胞肺癌外周血游离DNA及肿瘤细胞*EGFR*基因突变检测方法的研究进展

**DOI:** 10.3779/j.issn.1009-3419.2016.11.08

**Published:** 2016-11-20

**Authors:** 燕 徐

**Affiliations:** 100730 北京，中国医学科学院，北京协和医学院，北京协和医院呼吸内科 Department of Respiratory Medicine, Peking Union Medical College Hospital, Peking Union Medical College, Chinese Academy of Medical Sciences, Beijing 100730, China

**Keywords:** 肺肿瘤, 外周血, EGFR, Lung neoplasms, Plasma, EGFR

## Abstract

非小细胞肺癌（non-small cell lung cancer, NSCLC）是肺癌中最常见的类型。表皮生长因子受体（epidermal growth factor receptor, EGFR）酪氨酸激酶受体抑制剂（tyrosine kinases inhibitors, TKIs）是目前最重要的靶向治疗药物，对于有*EGFR*敏感突变的NSCLC，采用EGFR-TKIs治疗能取得明显的临床疗效；T790M突变是最常见的EGFR-TKIs耐药机制，通过对外周血进行*EGFR*基因突变检测，可以筛选出EGFR-TKIs治疗有效或治疗过程中对其产生耐药的患者。其定量分析不仅在肿瘤的早期诊断中具有重要意义，而且是疗效评价及随访的重要生物学指标。目前，用于检测外周血*EGFR*突变的方法有很多，其中以在数字PCR基础上建立起来的ddPCR灵敏度最高，实现了对样本的高通量检测，定量程度较其他方法更为精确，在临床基因诊断中具有广阔的应用前景。

## Abstract (en)

Non-small cell lung cancer (NSCLC) is the most common type of lung cancer. Epideral growth factor receptor tyrosine kinase inhibitors (EGFR-TKIs) are the most important treatments currently for advanced NSCLC patients harboring activating *EGFR* gene mutations, and achieve significant clinical efficacy. T790M mutation occurs in half of NSCLC patents with acquired EGFR-TKI resistance. Screening for *EGFR* gene mutations in histological and/or circulating tumor cell or DNA samples of NSCLC patients can identify patients who would have a response to EGFR-TKIs or acquire resistance during the treatment. Quantitative analysis of plasma *EGFR* mutation is of great importance not only in early diagnosis of tumors, but also in curative effect evaluation and for follow-up. However, a strict requirement is proposed on the detection technique because of low DNA content and the fragmentation of the genes. To date, many methods have been applied to detect cfDNA *EGFR* mutations, including sequencing, real-time PCR (RT-PCR), amplification refractory mutation system (ARMS), mutant-enriched PCR (ME-PCR), denaturing high-performance liquid chromatography (DHPLC), digital PCR, and droplet digital PCR (ddPCR). However, of all the methods above, ddPCR has the highest sensitivity, allows high throughput operation. In conclusion, the ddPCR has a lot of future promise in clinical gene diagnosis.

近年来，肺癌在癌症引起的死亡中已占据首位^[1]^。非小细胞肺癌（non-small cell lung cancer, NSCLC）是肺癌中最常见的类型，约占所有肺癌总数的80%，大多数NSCLC患者确诊时已处于晚期，失去了手术治疗或根治性放疗的机会，晚期NSCLC全身化疗中位生存期仅为8个月-10个月。目前，对于有明确驱动基因的NSCLC，分子靶向药物是治疗晚期NSCLC的重要策略。表皮生长因子受体（epidermal growth factor receptor, EGFR）酪氨酸激酶受体抑制剂（tyrosine kinases inhibitors, TKIs）是目前最重要的靶向治疗药物，对于有*EGFR*敏感突变的NSCLC患者，EGFR-TKIs治疗具有明显的临床疗效^[2-5]^。

部分癌细胞从原发肿瘤组织中脱落进入外周血、胸腔积液、唾液、粪便中，称为游离肿瘤细胞，游离肿瘤细胞发生凋亡或肿瘤细胞在原发肿瘤组织中凋亡坏死后均可释放DNA到外周血、胸腔积液、唾液、粪便等部位，这些DNA称为游离DNA（cell-free DNA, cfDNA）。研究^[6, 7]^证实，释放入外周血的游离肿瘤细胞或游离DNA可一定程度反映肿瘤基因表型。Karlovich等^[8]^曾采用不同检测方法反复试验后指出组织突变亦存在假阴性的可能，其原因可归结为肿瘤异质性，这在检测耐药突变T79M时更为突出，也间接证明了血浆代替肿瘤组织做为检测*EGFR*突变样本的优越性。

目前，用于检测外周血*EGFR*突变的方法有很多，包括测序法、实时荧光定量PCR（real-time PCR, RT-PCR）、扩增阻滞突变系统（amplification refractory mutation system, ARMS）、突变富集PCR（mutant-enriched PCR, ME-PCR）、变性高效液相色谱法（denaturing high performance 1iquid chromatography, DHPLC）及数字PCR等。以上方法各有其优缺点，本文将目前用于检测外周血*EGFR*基因突变的方法综述如下。

## 1 用于检测外周血*EGFR*基因突变的方法概述

### 1.1 测序法

测序法是目前检测基因突变最基础、应用最广、最直接、最准确的一种方法，不仅能检测已知突变，而且能发现新的突变，且费用不高，是目前公认的检测基因突变的金标准。该方法需要对待测序样品进行扩增、纯化、序列分析，过程繁琐，耗时较长，故在临床应用中存在一定的限制，不适用于大量临床样品分析。更重要的是测序法本身灵敏度不高，仅能检测突变含量在25%-30%以上的样本^[9-11]^。因此，对核酸含量较低的外周血来说，其检测效果不佳。由一代测序法即直接测序法发展而来的二代测序，其核心思想为边合成边测序，相比一代测序灵敏度及特异性有所提高，现有的技术平台包括Roche/454 FLX、Illumina、Solexa Genome Analyzer和Applied Biosystems SOLID system。

### 1.2 RT-PCR

RT-PCR于1996年由美国Applied Biosystems公司推出，该技术实现了PCR从定性到定量的飞跃，与常规PCR相比，其特异性更强，自动化程度高，目前已得到广泛应用。实时荧光定量PCR在常规PCR基础上加入荧光标记探针或相应的荧光染料来实现定量功能。其原理为：随着PCR反应的进行，PCR产物不断累积，荧光信号强度也等比例增加，每经过一个循环，荧光定量PCR仪收集一次荧光信号，这样就可以借助于荧光信号强度来监测PCR产物的变化，最后通过标准曲线对未知模板浓度进行定量分析。用荧光定量法检测目的基因并不需要检测突变基因组DNA具体含量，仅需检测样本是否具有扩增信号即可，并且PCR反应具有核酸扩增的高效性，可检测出微小突变。该方法可以检测出含量为1%以上突变^[12]^。但该技术的定量检测依赖于Ct值（cycle threshold），而Ct值会受扩增效率的影响，这在一定程度上限制了精确定量检测。

### 1.3 ARMS

1989年Newton等^[13]^在PCR的基础上，建立了AMRS，也称等位基因特异性扩增法（allele-specific amplification, ASA），或称等位基因特异PCR（allele specific PCR, AS-PCR）。该技术利用DNA聚合酶缺乏3’→5’外切酶活性，PCR引物的3’端末位碱基必须与其模板DNA互补才能有效扩增的原理，针对不同的已知突变，设计适当的引物以检测出突变基因。该法在设计引物时，在引物3’端设计一错配碱基，一个与野生DNA互补，一个与突变DNA互补，使之仅能与突变型或野生型互补而只扩增突变型或野生型基因。扩增产生的PCR产物可以通过凝胶电泳或是实时荧光定量PCR（real-time PCR）测定进行分析。ARMS法敏感性可达到1%^[14]^。蝎形探针扩增阻滞突变系统（Scorpions ARMS, SARMS）则是在扩增阻滞突变系统的基础上，引用了一种新型探针即Scorpions探针，由于Scorpions探针中序列特异性引物和探针在同一分子上，因此信号的产生特别快，其敏感性可达0.1%^[15]^。Cobas@是以ARMS为检测原理的伴随检验试剂盒，检测*EGFR*突变的敏感性达0.1%^[16]^。ARMS的不足之处在于只能检测已知突变。

### 1.4 ME-PCR

ME-PCR最早由Asano等^[11]^建立并用来检测*EGFR*基因突变。该方法通过两次PCR使突变基因得到富集，其灵敏度及特异性均较高，能从1×10^3^-1×10^4^个野生基因型拷贝中检测出一个突变基因^[17-19]^，且成本低廉，适合大医院开展肺癌患者常规追踪检测。其不足之处在于需要两次PCR，操作复杂，耗时长，容易污染而造成假阳性，只能检测一些常见的突变，而另一些较小的突变则检测不出，且检测通量较小。

### 1.5 DHPLC

DHPLC技术最早建立于1995年，近年来发展迅速，是在单链构象多态性和变性梯度凝胶电泳基础上发展起来的新杂合双链突变检测技术，可自动检测单碱基替代及小片段核苷酸的插入或缺失。该技术是根据受检DNA片段序列，经由特定软件分析选择维持一定的柱温，使受检DNA片段的双链达到部分变性，可被较低浓度的乙腈洗脱下来。由于错配的异源双链DNA与同源双链DNA的解链特征不同，在相同的部分变性条件下，异源双链DNA因有错配区的存在而更易变性，被色谱柱保留时间短于同源双链，从而在色谱图中表现为双峰或多峰的洗脱曲线，依据洗脱曲线可以直观分析判断结果。与测序法相比，DHPLC简单、快速，3 min就可完成一个样本的检测，不仅可用于已知突变的检测，还可以用于未知突变的扫描。在突变含量达到1.6%-6.25%以上时，DHPLC检测可行^[20, 21]^。其不足之处在于不能检测同源突变，也不能检测出突变的具体类型，结果判读容易出错，且当有多个片段需要检测时，由于有多个解链温度，需要多步检测，增加了工作量。

### 1.6 数字PCR

数字PCR的问世给基因分子检测带来了里程碑式的改变，其通过将样品大倍数稀释，使每个细分试样中所含有的待测分子数不超过1个，经PCR扩增后通过基因芯片逐个计数。这是一种绝对定量的方法，可以在大量野生型基因为背景时检测到含量极少的突变，无需校准。近年来，在数字PCR基础之上又衍生出如微乳滴磁珠PCR（BEAMing）、微数字PCR（microfluidics digital PCR）、微滴数字PCR（droplet digital PCR, ddPCR）等敏感性和特异性更高、定量更精确、效率也更高的检测方法。BEAMing2003年推出，该方法结合了数字PCR与流式技术，通过将引物结合在磁珠表面，再将单个磁珠与目标分子包裹在微乳液滴中进行PCR扩增，将野生型和突变型目标分子在磁珠表面进行复制，扩增结束后进行破乳，再利用流式细胞技术进行荧光计数。其灵敏度高，适合用于低概率的等位基因突变分析，其检测*EGFR*突变的敏感性达0.02%^[22]^。微数字PCR是在数字PCR的基础上结合了基因芯片技术，有效避免了交叉污染，操作时间短，可在2 h内（仅需20 min的人工操作时间）完成12个样品的同步检测，检测灵敏度达0.1%^[23]^。但由于目前基因芯片技术发展还不甚成熟，其在临床的实际应用程度仍然有限。Bio-Rad公司于2011年推出了ddPCR系统^[24]^，该技术在传统的PCR扩增前对样品进行微滴化处理，可以将每份样本分成20, 000个微滴，而后进行PCR扩增，根据泊松分布原理及阳性微滴的个数与比例，可得出靶分子的起始拷贝数或浓度，是一种核酸分子绝对定量技术，其检测敏感性高达0.001%^[15, 24, 25]^。

## 2 外周血*EGFR*基因突变检测

测序法目前被公认为是检测基因突变的金标准，但仅能检测突变含量在25%-30%以上的样本，外周血*EGFR*突变检出率波动也较大，约在5.1%-37.0%^[12, 14, 26]^。因此，对于外周血这类cfDNA及肿瘤细胞含量少、野生型占比高的样本来说，测序法并不是最优选择。ARMS法敏感性可达0.1%，但只能检测已知突变。日本学者Kimura等^[26, 27]^在其两项研究中用测序法和SARMS法分别检测了血清*EGFR*的两个热点突变，发现采用SARMS法得到的突变率明显高于测序法（48.1% *vs* 37.0%），其灵敏度为50.0%-75.0%，特异度85.7%-97.1%，一致率达72.7%-92.9%；值得一提的是，SARMS法检测得到的血清*EGFR*敏感突变阳性的患者在接受靶向治疗后，其中位无疾病进展生存期（progression-free survival, PFS）、总生存期（overall survival, OS）明显长于突变阴性患者，显示出敏感突变与靶向治疗反应的一致性，而采用测序法则并未得到这一结论。Maheswaran等^[28]^曾选取20例配对的肿瘤组织及外周血游离肿瘤细胞，其肿瘤组织已知均含有*EGFR*敏感突变，采用SARMS法对外周血游离肿瘤细胞进行检测，共检测出19例与肿瘤组织相一致的突变，检出率达95.0%；该研究同时比较了12例配对外周血游离肿瘤细胞和血浆cfDNA分别作为样本时在检测*EGFR*突变方面的优劣，结果提示以游离肿瘤细胞为样本时能更有效的检测出突变（92% *vs* 33%, *P*=0.009）。在IPASS^[29]^研究中的亚组分析中，曾采用SARMS法对86例血清cfDNA进行*EGFR*基因突变检测，其灵敏度为43.1%，特异度为100.0%，一致率为66.3%。Douillard等^[30]^采用SARMS法检测了652例高加索人种的血浆cfDNA *EGFR*基因突变，其灵敏度为65.7%，特异度为99.8%，检测一致率达到94.3%。ME-PCR灵敏度及特异性较高，能从2, 000个野生拷贝中检测到一个突变基因，而普通PCR仅能从10个野生拷贝中检测到一个突变基因，ME-PCR明显优于普通PCR及测序法^[11, 31, 32]^。He等^[12]^采用ME-PCR对血浆*EGFR*基因突变进行检测，得到的突变率为49.3%（66/134），其敏感性明显高于测序法（33/60, 55.0% *vs* 11/60, 18.3%, *P* < 0.001），与配对肿瘤组织的检测一致率达94.4%（17/18）。Zhao等^[33]^采用ME-PCR法对111例血浆样本进行突变分析，灵敏度为35.6%，特异度95.5%，与组织检测结果一致率为71.2%。Bai等^[21]^随机选取了230例进展期NSCLC，采用DHPLC得到的血浆*EGFR*突变率为34.3%，灵敏度为81.8%，特异度为90.8%，与组织突变结果一致率为87.0%。Yung等^[23]^采用数字PCR对31例血浆进行突变检测，与测序法测得的组织突变结果相比，其灵敏度为91.7%，特异度100.0%，一致率高达96.8%；同时，该研究利用数字PCR的定量功能发现对EGFR-TKIs反应较好的患者，其血浆突变基因含量会随着治疗的进行而降低，提示我们对*EGFR*突变进行定量分析可以指导临床医生做出更好的治疗决策。Zhu等^[34]^将突变DNA稀释至不同浓度，测得ddPCR敏感性达0.04%；与ARMS法检测得到的组织突变结果比较，其检测血浆cfDNA灵敏度和特异度均较高，分别为81.8%和98.4%。本文作者亦就ddPCR检测血浆cfDNA *EGFR*两个热点突变的灵敏度、特异度及与组织结果的一致性进行了相关研究，样本例数充足，共检测样本215例，得到的结果分别为61.3%、96.7%、81.4%，与Oxnard等^[25]^得到的结果较为一致。Thress等^[35]^在其研究中详细比较了Cobas@、BEAMing及ddPCR检测*EGFR*两种常见敏感突变的优劣，不管是灵敏度、特异度还是与组织突变结果的一致性方面，三种检测方法均取得了另人满意的结果。其中以BEAMing灵敏度最高，达93%以上，Cobas@最低，但也在86%以上，ddPCR居中，为90%；特异性方面，Cobas@与ddPCR均达到了100%，BEAMing在93%以上；与组织突变的一致率方面，均能达到90%以上，其中以ddPCR最高，达到了97%。Karlovich等^[8]^在其试验中同样采用Cobas@检测血浆ctDNA *EGFR*敏感突变，其特异度同样达到100%，但灵敏度及与组织突变一致率较低，不到80%。表 1列出了各研究采用不同方法检测外周血*EGFR*两种最常见敏感突变数据特点。

**1:** 不同方法检测外周血*EGFR*敏感突变结果汇总 Characteristics of different methods evaluating *EGFR* activating mutation in peripheral blood

First author	Country	Year	Detection methods	No. of samples	Sample type	Mutation rate (%)	Sensitivity (%)	Specificity (%)	Consistentcy rate (%)
Kimura H^[26]^	Japan	2006	Sequencing	27	Serum	37.0	-	-	-
Liu Y^[14]^	China	2011	Sequencing	78	Plasma	5.1	-	-	-
Kimura H^[26]^	Japan	2006	S-ARMS	27/11	Serum	48.1/27.3	-/50.0	-/85.7	-/72.7
Kimura H^[27]^	Japan	2007	S-ARMS	42	Serum	16.7	75.0	97.1	92.9
Maheswaran S^[28]^	USA	2008	S-ARMS	20	Peripheral blood CTC	-	-	-	95.0
Goto K^[29]^	Japan	2012	S-ARMS	86	SerumcfDNA	25.6	43.1	100	66.3
Douillard JY^[30]^	Europe	2014	S-ARMS	652	Plasma cfDNA	10.7	65.7	99.8	94.3
He C^[12]^	China	2009	ME-PCR	134/18	Plasma	49.3/50.0	-/100.0	-/90.0	-/94.4
Zhao X^[33]^	China	2013	ME-PCR	111	Plasma	17.1	35.6	95.5	71.2
Bai H^[21]^	China	2014	DHPLC	230	Plasma	34.3	81.8	90.8	87.0
Yung TK^[23]^	China	2009	Digital PCR	31	Plasma	35.5	91.7	100.0	96.8
Oxnard GR^[25]^	USA	2014	ddPCR	46	Plasma cfDNA	30.4	66.7	100.0	84.8
Zhu G^[34]^	China	2015	ddPCR	86	Plasma cfDNA	43.0	81.8	98.4	93.0-94.2
Thress KS ^[35]^	USA	2015	cobas^@^	38	Plasma ctDNA^#^	-	86	100	89
Thress KS^[35]^	USA	2015	ddPCR	38	Plasma ctDNA^#^	-	90	100	97
Thress KS^[35]^	USA	2015	BEAMing	38	Plasma ctDNA^#^	-	93	93	95
Karlovich C^[8]^	USA	2016	cobas^@^	99	Plasma ctDNA^#^	55.6	73.3	100	79.8
ctDNA: circulating tumor DNA.									

## 3 外周血T790M突变检测与EGFR-TKIs获得性耐药

在EGFR-TKIs治疗过程中，所有患者最终均会对EGFR-TKIs产生耐药，其中T790M突变为最常见的耐药机制，约占所有耐药机制的47%-66%^[36-38]^。已有文献^[25, 39]^报道，T790M突变在外周血中的出现要早于影像学进展，最早可在影像学进展前16周在外周血中检测到T790M突变的存在。及时对患者的T790M突变状态进行动态检测便于临床工作者做出更好的医疗决策，这在实际临床工作中对T790M的监测造成了很大限制，并且肿瘤组织有很大的异质性，而以外周血为标本检测T790M突变相较肿瘤组织则具备了很多优势。Sorensen等^[40]^采用ARMS法对T790M连续监测发现，其最早在临床出现明显进展前344天就已经出现在血浆DNA中。Ishii等^[41]^将数字PCR用于EGFR-TKIs耐药后T790M的检测，其灵敏度达81.8%，特异度85.7%，与组织检测结果一致率为83.3%。Thress等^[35]^选取38例样本详细比较了Cobas@、ddPCR、BEAMing在检测血浆ctDNA T790M突变灵敏度、特异度及与组织的一致率。ddPCR与BEAMing灵敏度及与组织突变状态一致率取得了令人满意的结果，在70%以上，Cobas@灵敏度仅为41%，与肿瘤组织一致率不足60%。而在Karlovich^[8]^的研究中，Cobas@检测T790M的灵敏度及与组织突变的一致率均有所提高，分别为63.3%和86.3%。Thress等^[35]^进一步选取了72例样本对比了Cobas@与BEAMing检测血浆ctDNA的优劣，二者灵敏度均另人满意，但BEAMing稍高于Cobas@（81% *vs* 73%），但BEAMing检测T790M的特异性不及Cobas@（58% *vs* 67%）；二者检测T790M的一致率高达90%；该研究同时指出，同样经过AZD9291治疗的患者，采用Cobas@测得的组织T790M突变阳性患者的客观缓解率与经同样方法测得的血浆阳性患者一致。以上均说明了Cobas@和BEAMing可以用于检测血浆是否含有T790M突变。从以上各学者的研究中可以看出，与非数字化试剂盒相比，数字化试剂盒对游离DNA T790M的检测更具优势，其灵敏度更高。Zheng等^[39]^采用ddPCR对EGFR-TKIs耐药后的25例血浆ctDNA进行T790M突变检测，灵敏度81.%，特异度100.0%，组织突变一致率88.0%。

## 4 总结

如何快速简便高效的筛选出*EGFR*突变是当下基础科研人员及临床医生共同关心的问题。目前还没有一种完全适用于临床的标准统一的检测外周血cfDNA及游离肿瘤细胞*EGFR*基因突变的方法。

RT-PCR、AMRS、ME-PCR、DHPLC、数字PCR等方法与测序法相比，其灵敏度及特异性均得到很大的提高，RT-PCR、ARMS、ME-PCR及数字PCR更实现了对突变的定量检测，这对于靶向药物治疗后疗效评估是十分必要的。RT-PCR法单次反应只能对1个或少数突变进行检测，无法实现高通量。ARMS法重复性好，其敏感性为1%，而在结合Scorpions探针后其敏感性可达0.1%。Kimura等^[26]^以血浆为样本采用ARMS进行*EGFR*突变检测时，发现L858R的检出率仅为3.7%，而对E19 del的检出率可达44.4%，提示在检测突变含量较少，而大量野生型基因作为背景的样本时，ARMS对于点突变的检测可能不太灵敏。ME-PCR需要两次PCR，而且需要酶切，操作复杂，耗时长，容易污染而造成假阳性，只能检测一些常见的突变，而另一些较小的突变则检测不出。DHPLC可以用于未知突变的扫描，但不能检测出突变类型，结果判读容易出错，且当有多个片段需要检测时，由于有多个解链温度，需要多步检测，增加了工作量，其灵敏度与其他方法相比较低，为1.6%-6.25%。

目前，各学者就不同检测方法的性能都在进行相关研究，但得到的结果却不太一致，甚至大相径庭。笔者认为，除检测方法本身的特性外，以外周血游离肿瘤细胞或cfDNA为样本进行*EGFR*基因突变检测过程中还受很多其它因素影响，包括样本质量、DNA含量、DNA的提取、样本的临床病理特征如病理类型、肿瘤分期、肿瘤分化程度等等。Maheswaran等^[28]^曾建立了CTC-chip设备，能够分离、定量、从血样中分析游离肿瘤细胞，改善样本提取质量，从而可以提高突变检测效率。Oxnard等^[25]^通过测量长散在核元件（long interspersed element 1, LINE-1）浓度来评估cfDNA的质量和数量，研究表明，当样本LINE-1低于设定的中位浓度时，其检出*EGFR*突变的灵敏度只有50%，而高于LINE-1中位浓度时，其突变检出灵敏度可提高至81%。Zhao等^[33]^在其研究中指出，以晚期NSCLC患者的血浆为样本进行检测时，其敏感性及与组织的一致率明显高于偏早期患者的血浆样本检测结果；而且，肿瘤分化较差患者的血浆检测结果，其敏感性、与肿瘤组织的一致率明显高于高分化肿瘤患者的血浆检测结果。越来越多的学者将两种方法相结合以更好地利用各方的优势。有学者将AMRS法与实时荧光定量PCR结合以避免电泳带来的有毒操作；将测序法结合构象敏感凝胶电泳（conformation-sensitive gel electrophoresis, CSGE）以提高其灵敏度^[42, 43]^。

数字PCR及在此基础上建立起来的BEAMing、微数字PCR、ddPCR及伴随诊断试剂盒Cobas@，实现了对样本的高通量定量检测，尤其是ddPCR，其灵敏度极高，可检测出含量为0.001%的突变，而且省时，3 h可以对96份样本进行结果判读，且定量程度较其他方法更为精确，这对于靶向药物治疗后疗效评估及耐药机制研究来说十分重要，相信其在临床基因诊断、第三代EGFR-TKIs作用机制研究中将会呈现无可比拟的优越性。
